# Ubrogepant Plasma and Cerebrospinal Fluid Exposures in Participants With a History of Migraine: Findings From a Phase 1b Open‐Label Trial

**DOI:** 10.1111/cts.70560

**Published:** 2026-04-21

**Authors:** Ramesh R. Boinpally, Joel M. Trugman

**Affiliations:** ^1^ Clinical Pharmacology, AbbVie Inc. North Chicago Illinois USA; ^2^ Clinical Development, AbbVie Inc. North Chicago Illinois USA

**Keywords:** central nervous system, cerebrospinal fluid, gepant, ubrogepant

## Abstract

Ubrogepant (Ubrelvy), a CGRP receptor antagonist, is approved for acute treatment of migraine with or without aura in adults. Because ubrogepant treats a neurological condition, it is of interest to know central nervous system exposure. Ubrogepant concentrations in plasma and cerebrospinal fluid (CSF) were measured in participants with a history of migraine. This analysis included a subset of participants with a ≥ 1 year history of migraine who were enrolled in a Phase 1b multi‐center, open‐label trial and who underwent a single CSF collection via lumbar puncture. All participants received a single oral dose of 100 mg ubrogepant. Plasma and CSF ubrogepant concentrations were measured using LC–MS/MS at pre‐defined post‐dose timepoints (plasma: 0–24 h; CSF: 2 or 4 h). Ubrogepant CSF/plasma concentration ratios at 2 and 4 h were determined. Ubrogepant safety and tolerability were evaluated through reporting of treatment‐emergent adverse events (TEAEs; ≤ 30‐days after dosing), physical examination, ECG, laboratory testing, and vital sign monitoring. A total of 8 participants were included. Mean (±SD) age was 34.5 ± 9.58 years and 4 participants (50%) were male. At 2 h, mean (±SD) CSF and plasma ubrogepant concentrations were 1.1 ± 0.8 and 354 ± 163 ng/mL, respectively (CSF/plasma ratio: 0.36% ± 0.29%); at 4 h, 2.3 ± 1.9 and 218 ± 84 ng/mL, respectively (CSF/plasma ratio: 0.94% ± 0.64%). No serious TEAEs, deaths, or significant changes in laboratory values, vital signs, or ECGs occurred. Because ubrogepant half maximal effective concentration (EC_50_) has been estimated at ~1.4 ng/mL, ubrogepant could act both centrally and peripherally to relieve migraine symptoms. ClinicalTrials.gov, NCT04179474 (registration date: 27 November 2019).

## Study Highlights

**What Is the Current Knowledge on the Topic?:**

Ubrogepant, a small molecule CGRP receptor antagonist, is approved for acute treatment of migraine in adults. Systemic exposures following dosing have been well‐characterized but central nervous system (CNS) exposure has not been previously studied.

**What Question Did This Study Address?:**

The current study directly examined plasma and CSF concentrations following administration of a single 100 mg ubrogepant dose.

**What Does This Study Add to Our Knowledge?:**

CSF ubrogepant concentration was 2.26 ng/mL 4 h after a single 100 mg dose (CSF/plasma ratio: 0.94%), which was well above the half maximal effective concentration (EC_50_) of ~1.4 ng/mL. Therefore, ubrogepant can cross the blood–brain barrier to a limited extent and likely acts both centrally and peripherally to relieve migraine symptoms.

**How Might This Change Clinical Pharmacology or Translational Science?:**

This study provides further insight on ubrogepant clinical pharmacology in the CNS and provides evidence that CNS CGRP inhibition likely plays a role in relieving migraine symptoms.

## 1 Introduction

Migraine affects over 1 billion people worldwide [1], negatively impacting nearly all aspects of quality of life [2, 3, 4]. Calcitonin gene‐related peptide (CGRP) has been implicated in migraine pathogenesis, and CGRP‐receptor antagonists (gepants) have well‐established efficacy for both the acute treatment and prevention of migraine [5, 6]. Ubrogepant is an orally administered, highly selective, small molecule, CGRP‐receptor antagonist approved for the acute treatment of migraine with or without aura in adults [7]. Because the drug improves central nervous system symptoms of migraine, including pain, photophobia, and phonophobia [8, 9], it is of interest to know if the drug has a direct impact on the central nervous system or if it primarily acts peripherally. Therefore, ubrogepant concentrations in plasma and cerebrospinal fluid (CSF) were measured in a subset of patients in an ubrogepant Phase 1b clinical trial (NCT04179474) [10]. Here, we report ubrogepant plasma and CSF concentrations, along with the plasma/CSF ratios, in adults (18–50 years old) with a ≥ 1‐year history of migraine.

## 2 Methods

This study reports plasma and CSF ubrogepant concentration data obtained as part of a phase 1b clinical trial in adult patients (18–50 years of age) with a history of migraine (NCT04179474). The Bio‐Kinetic Clinical Applications Institutional Review Board (IRB; Springfield, MO; IRB protocol number: 17419, date: 03 September 2019) and the Advarra IRB (Columbia, MO; IRB protocol number: Pro00038133, date: 22 August 2019) approved the study protocol, informed consent forms, and recruitment materials before patient enrollment. The study was conducted in accordance with the International Council for Harmonization guidelines, applicable regulations, and the Declaration of Helsinki. All patients provided written informed consent before screening.

### 2.1 Participants and Study Design

This Phase 1b, 2‐part, multi‐center, fixed‐sequence, open‐label, randomized, clinical trial included male and female participants who were 18–50 years of age and had ≥ 1‐year history of migraine (study inclusion/exclusion criteria provided in Table S1). This analysis includes a subset of participants who consented to have a single CSF sample collected. The trial had two study sites but all participants in the CSF collection subset were from a single site.

Full study design and study procedures have been fully described elsewhere [10]. Briefly, all participants received a single oral dose of 100 mg ubrogepant (Day 1) under fasting conditions and had venous blood samples collected for pharmacokinetic analysis. A subset of participants consented to also have a CSF sample collected for ubrogepant concentration measurement. Blood samples were collected via venipuncture pre‐dose and at 0.5, 1, 1.5, 2, 3, 4, 5, 6, 8, 12, 14, and 24 h post‐dose; a single CSF sample was collected via lumbar puncture at either 2 or 4 h post‐dose. Ubrogepant safety was assessed through treatment‐emergent adverse event (TEAE) reporting (MedDRA version 22.1 coding; any adverse event ≤ 30 days after dosing) and ECG parameter, clinical laboratory testing (serum chemistry), and vital sign (blood pressure, pulse rate) monitoring.

### 2.2 Ubrogepant Concentration Measurements

Ubrogepant concentrations were measured in plasma using a validated liquid chromatography tandem mass spectrometry (LC–MS/MS) method and in CSF using a qualified LC–MS/MS method [10]. Briefly, blood samples were centrifuged within 30 min of collection (≥ 2500 g for 10 min at ~4°C). Plasma and CSF samples were collected, flash‐frozen, and kept at approximately −20°C until analysis. Samples underwent protein precipitation extraction followed by reverse‐phase high‐performance liquid chromatography for ubrogepant. Ubrogepant was detected using a quadrupole mass spectrometer (Turbo V ion source with electrospray ionization probe, positive ionization mode). The assay was linear over the range of 1–1000 ng/mL and met current regulatory standards of precision and accuracy within 15%.

### 2.3 Data Analysis

The current pharmacokinetic analysis included all participants who had at least one CSF ubrogepant concentration measurement. Ubrogepant plasma pharmacokinetic parameters were determined from plasma concentration data using noncompartmental analysis. Descriptive statistics (arithmetic mean, standard deviation, relative standard deviation, maximum, minimum) were reported for ubrogepant CSF concentrations at each nominal time point examined on Day 1. The ubrogepant CSF/plasma concentration ratio (expressed as percent) was also calculated for each timepoint examined and reported using descriptive statistics (arithmetic mean, standard deviation, relative standard deviation, maximum, minimum). Pharmacokinetic analysis, concentration‐time data and PK parameter summaries, and PK parameter statistical analysis were performed using Phoenix WinNonlin software (version 8.0, Certara, Radnor, PA). Actual sampling times were used in PK parameter value calculations; nominal sample times were used in descriptive statistics calculations. Concentration data below LLOQ was input as zero.

## 3 Results

A total of 8 participants were included in CSF‐related analyses. Participant characteristics are described in Table 1. Overall, a single 100 mg oral ubrogepant dose was well‐tolerated in this small study of participants with a history of migraine. Six of 8 participants (75.0%) experienced ≥ 1 TEAE, all of which were common following lumbar puncture (Table 1; Table S2). No serious TEAEs, deaths, or significant changes in clinical laboratory values, vital signs, or ECG parameters occurred.

**TABLE 1:** Participant characteristics, concomitant medications, and safety summary following administration of 100 mg ubrogepant and lumbar puncture for CSF sample collection.

	(*N* = 8)
Participant characteristic	
Age, years, mean ± SD	34.5 ± 9.58
Male, *n* (%)	4 (50%)
BMI, kg/m^2^, mean ± SD	28.31 ± 5.19
Race, *n* (%)	
Black or African American	5 (62.5%)
White	3 (37.5%)
Ethnicity, *n* (%)	
Hispanic or Latino	3 (37.5%)
Concomitant medications, *n* (%)	
Lidocaine 1%, subQ (local anesthesia for LP)	8 (100%)
Midazolam, IV (sedation for LP)	8 (100%)
Naproxen sodium, oral (post‐LP headache)	2 (25.0%)
Acetaminophen, oral (post‐LP back pain)	1 (12.5%)
Normal saline, IV (LP site pain)	1 (12.5%)
Excedrin migraine, oral (migraine headache)	1 (12.5%)
Sumatriptan, oral (migraine headache)	1 (12.5%)
TEAE description^a^	
≥ 1 TEAE, *n* (%)	6 (75.0%)
Lumbar puncture site pain	5 (62.5%)
Post‐lumbar puncture syndrome	2 (25.0%)
Neck pain	1 (12.5%)
Peripheral neuropathy	1 (12.5%)
Vomiting	1 (12.5%)
≥ 1 Serious TEAE, *n* (%)	0
Deaths	0

Abbreviations: CSF, cerebrospinal fluid; IV, intravenous; LP, lumbar puncture; subQ, subcutaneous; TEAE, treatment‐emergent adverse event (≤ 30 days after ubrogepant dosing).

^a^ Coded using MedDRA v22.1 preferred terms.

### 3.1 Ubrogepant Plasma and Cerebrospinal Fluid Exposures

**FIGURE 1:**
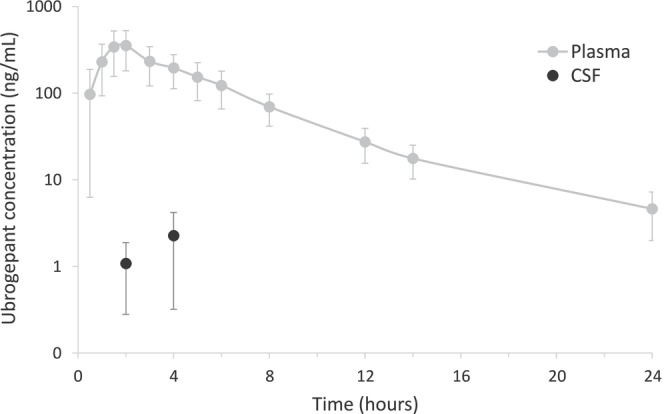
Mean ubrogepant concentration‐time profile in plasma (*N* = 8) and CSF (*N* = 4 at each time point) following administration of a single oral dose of 100 mg ubrogepant in participants with a history of migraine. Mean (±SD) CSF/plasma ratio was 0.36% ± 0.29% at 2 h and 0.94% ± 0.64% at 4 h. Error bars represent SD. CSF, cerebrospinal fluid; SD, standard deviation.

All 8 participants had a single CSF (2 or 4 h after dosing) and multiple plasma ubrogepant concentration measurements. Mean maximum plasma ubrogepant concentration (C_max_) was 354 ± 163 ng/mL, observed 2 h after dosing, falling to 218 ± 84 ng/mL 4 h after dosing (*N* = 8; Figure 1). As expected, ubrogepant concentration was lower in CSF than in plasma. At 2 and 4 h, mean (±SD) CSF ubrogepant concentrations were 1.1 ± 0.8 (CSF/plasma ratio: 0.36% ± 0.29%) and 2.3 ± 1.9 ng/mL (CSF/plasma ratio: 0.94% ± 0.64%), respectively (*N* = 4 at each timepoint; Figure 1).

## 4 Discussion

The current study confirms that a small proportion of orally administered ubrogepant reached the central nervous system. Ubrogepant mean concentration in the CSF was 2.3 ± 1.9 ng/mL (~1% of plasma concentration) at 4 h after administration of a single 100 mg oral ubrogepant dose. Ubrogepant is 87% plasma protein bound [7], which may have played a role in the relatively low proportion of ubrogepant crossing the BBB. However, in vitro studies showed that ubrogepant has a high binding affinity for human CGRP receptors (inhibitory constant: ~0.04 ng/mL [0.07 nM]) and low nanomolar activity (cAMP IC_50_: ~0.1 ng/mL [0.19 nM] in the presence of human serum) [11]. Further, prior pharmacodynamic assessment demonstrated an ubrogepant half maximum effective concentration (EC_50_) in humans of ~1.4 ng/mL (2.6 nM) based on a capsaicin‐induced dermal vasodilation (CIDV) model [11]. Though this EC_50_ is representative of ubrogepant's ability to inhibit CGRP‐related blood flow in the periphery, ubrogepant CSF concentration was high enough within 4 h of administration to directly and meaningfully inhibit CGRP receptors. Together, these in vitro and in vivo findings suggest that ubrogepant's anti‐migraine effect stems, at least in part, from its action on CGRP receptors both outside and inside the CNS. Ubrogepant is a p‐glycoprotein (P‐gp) substrate and one may argue that its brain tissue concentration could be lower than CSF levels. However, ubrogepant CSF/plasma, brain/plasma and brain/CSF exposure ratios in rat were 0.0027, 0.0128 and 4.8293, respectively (based on area under the concentration‐time curves [AUC]; unpublished data), providing evidence that brain ubrogepant concentration in humans could be higher than the measured CSF levels. Therefore, ubrogepant central nervous system exposure is likely high enough in most patients to directly and meaningfully inhibit brain CGRP activity.

Gepants were expected to have a moderate ability to cross the blood–brain‐barrier (BBB) due to their low molecular weight but evidence of BBB transport or a direct effect on the central nervous system is limited. An ubrogepant pharmacodynamic study in rhesus monkeys showed 0%–16% CGRP receptor occupancy at plasma concentrations of 53–203 nM (29–112 ng/mL; monkey CIDV EC_50_: 3.19 nM) [11]. A similar study of [^11^C]atogepant showed ≤ 25% brain CGRP receptor occupancy at plasma concentrations of ≤ 229 nM (≤ 126 ng/mL; monkey CIDV EC_50_: 1 nM) [12]. Further, a PET imaging study on radiolabeled telcagepant, a first‐generation gepant (not approved for marketing), showed 4%–10% CGRP receptor occupancy following oral administration of a therapeutic dose (140 mg) in healthy participants [13]. These findings suggest limited transport of gepants across the BBB. However, the observed ubrogepant CSF concentration was well above the EC_50_ in most patients in the current study suggesting that ubrogepant likely acts directly on the central nervous system.

Central nervous system action has been investigated for other classes of migraine treatments. Triptans have limited transport across the BBB but studies have demonstrated sufficient concentration to stimulate 5‐HT receptors in the central nervous system as well as brain 5‐hydroxytryptamine (5‐HT) receptor occupancy in humans [14]. Further, evidence in rats suggests a high level of targeted 5‐HT receptor occupancy in the trigeminal ganglion [15] but the impact of this peripheral nervous tissue binding on the central nervous system is not well‐understood [15, 16]. Verapamil, a calcium channel blocker, is used as a prophylaxis for cluster headaches. It has limited access to the central nervous system but is thought to act after it crosses the BBB [17]. Non‐steroidal anti‐inflammatory agents possibly influence trigeminovascular system activation, a key pain‐generating component of migraine, through cyclo‐oxygenase (COX) inhibition [18]. Ibuprofen, flurbiprofen, and indomethacin have been shown to rapidly cross the BBB in rats [19] and reduce migraine pain via both peripheral tissue and central nervous system actions [20].

This study had two main limitations. First, CNS ubrogepant pharmacodynamic activity was assumed based on demonstrated peripheral CIDV inhibition [11]. Because of obvious challenges in directly measuring CGRP inhibition in the human CNS, change in CIDV was developed as a proxy [21]. However, CIDV is particularly attractive for measuring the pharmacological activity of gepants because it is, in large part, mediated by CGRP and CGRP inhibition is the only mechanism shown to antagonize it [21]. Second, ubrogepant CSF concentration may not have reached its maximum at 4 h post‐dose, as supported by the observation that the percent of patients achieving pain freedom continued to increase 8 h after dosing [8]. However, any further increase in ubrogepant CNS concentration would only strengthen the evidence supporting a direct pharmacological effect of ubrogepant on the CNS.

In conclusion, results of the current study suggest that ubrogepant, a small molecule CGRP receptor antagonist, crosses the BBB in humans, although to a limited extent. Given ubrogepant's potency and that CSF concentrations were above its EC_50_, central nervous system concentrations were theoretically high enough for the drug to pharmacologically act directly on CGRP receptors in the central nervous system. Therefore, ubrogepant could act both centrally and peripherally to relieve migraine symptoms.

## Author Contributions

R.R.B. and J.M.T. wrote the manuscript; R.R.B. designed the research; R.R.B. and J.M.T. performed the research; J.M.T. and R.R.B. analyzed the data.

## Funding

This study was funded by Allergan Inc. (acquired by AbbVie in May 2020). The study sponsor participated in study design, research, analysis, data collection, interpretation of data, reviewing, and approval of the publication. No honoraria or payments were made for authorship. Article publication fees were funded by AbbVie.

## Conflicts of Interest

R.R.B. is an employee of AbbVie Inc. and may hold AbbVie stock, stock options, and/or patents on ubrogepant. J.M.T. is a former employee of AbbVie and may hold AbbVie stock and/or patents on ubrogepant.

## Supporting information


**Table S1:** Study enrollment criteria.
**Table S2:** Listing of TEAEs in participants who received a single dose of 100 mg ubrogepant and underwent a single post‐dose collection of CSF.
