# Supplementary material for: Ubrogepant Plasma and Cerebrospinal Fluid Exposures in Participants With a History of Migraine: Findings From a Phase 1b Open‐Label Trial
Source: Clin Transl Sci. 2026 Apr 21;19(4):e70560. doi: 10.1111/cts.70560 (PMC13097550; doi:10.1111/cts.70560)
Supplement: Supplementary file 1 — Table S1: Study enrollment criteria. Table S2: Listing of TEAEs in participants who received a single dose of 100 mg ubrogepant and underwent a single post‐dose collection of CSF. [file CTS-19-e70560-s001.docx]

**SUPPLEMENT TO:**

**Ubrogepant plasma and cerebrospinal fluid exposures in participants with a history of migraine: findings from a Phase 1b open-label trial**

Ramesh R. Boinpally^1^ and Joel M. Trugman^2^

^1^Clinical Pharmacology, AbbVie Inc., North Chicago, Illinois, USA

^2^Clinical Development, AbbVie Inc., North Chicago, Illinois, USA

**Supplemental Contents:**

**Table S1.** Study enrollment criteria.

**Table S2.** Listing of TEAEs in participants who received a single dose of 100 mg ubrogepant and underwent a single post-dose collection of CSF

**Table S1.** Study enrollment criteria.

| **Inclusion criteria** | |
| --- | --- |
| Age | - 18-50 years of age (inclusive) at the time of informed consent signing |
| Participant and migraine characteristics | - ≥1 year history of migraine with/without aura consistent with a diagnosis according to the International Classification of Headache Disorders, 3rd edition (ICHD-3, 2018) - By history, the participant’s migraines typically last between 4 and 72 hours if untreated or treated unsuccessfully and migraine episodes are separated by at least 48 hours of headache pain freedom - History of ≥2 migraine attacks/month in the 2 months prior to Screening - Sitting pulse rate ≥45 bpm and ≤100 bpm during the vital sign assessment at the Screening Visit - Negative test results for benzoylecgonine (cocaine), methadone, barbiturates, amphetamines, benzodiazepines, cannabinoids, opiates, and phencyclidine at the Screening Visit and Day -1 unless explained by concomitant medication use (e.g., opioids prescribed for migraine pain) |
| Weight and BMI | - BMI ≥18 kg/m^2^ and ≤40 kg/m^2^ at the Screening Visit |
| Sex | - Male and/or female |
| Contraceptives: male | - Male participant willing to minimize the risk of inducing pregnancy for the duration of the clinical study and follow-up period - A male participant must agree to use contraception during the intervention period and for ≥93 days after the last dose of study intervention and refrain from donating sperm during this period |
| Contraceptives: female | - Female participants willing to minimize the risk of pregnancy for the duration of the clinical study and follow-up period - A female participant is eligible to participate if she is not pregnant (negative serum pregnancy test at the Screening Visit and a negative serum or urine pregnancy test on Day -1) and has ≥1 of the following:   - Not a woman of child-bearing potential   - A woman of child-bearing potential who agrees to follow contraceptive guidance during the study intervention period and for ≥33 days after the last dose of study intervention |
| Informed consent | - Capable of giving signed informed consent, which includes compliance with study requirements and restrictions. Written documentation has been obtained in accordance with relevant country and local privacy requirements, where applicable. |
| Other | - Able, as assessed by the investigator, and willing to follow study instructions and likely complete all required study visits. |
| **Exclusion criteria** | |
| Medical conditions and history | - Difficulty distinguishing migraine headache from tension-type or other headaches - History of migraine aura with diplopia or impairment of level of consciousness, hemiplegic migraine, or retinal migraine as defined by ICHD-3 - Had a current diagnosis of new persistent daily headache, trigeminal autonomic cephalgia (e.g., cluster headache), or painful cranial neuropathy as defined by ICHD‑3 |

**Table S1** (continued)**.** Study enrollment criteria.

| **Exclusion criteria** (continued) | |
| --- | --- |
| Medical conditions and history (continued) | - Required hospital treatment of a migraine attack ≥3 times in the 6 months prior to screening - Had a chronic non-headache pain condition requiring daily pain medication (with the exception of pregabalin) - Clinically significant cardiovascular or cerebrovascular disease per the investigator’s opinion including, but not limited to: - Clinically significant ischemic heart disease (e.g., unstable angina pectoris) - Clinically significant cardiac rhythm or conduction abnormalities (e.g., atrial fibrillation, second- or third-degree heart block) or risk factors for Torsade de Pointes (e.g., heart failure, hypokalemia, bradycardia) - Myocardial infarction, transient ischemic attack, or stroke within 6 months prior to screening - Heart failure defined as New York Heart Association functional classification system, Class III or IV - Any clinically significant hematologic, endocrine, pulmonary, renal, hepatic, gastrointestinal, or neurologic disease   - History of such disease but condition was stable for >1 year prior to screening and was judged by the investigator as not likely to interfere with the participant’s participation in the study, the participant may have been included   - Participants on dialysis for renal failure were excluded - In the opinion of the investigator, other confounding pain syndromes, confounding psychiatric conditions, dementia, epilepsy or other significant neurological disorders other than migraine - History of malignancy in the 5 years prior to screening, except for adequately treated basal cell or squamous cell skin cancer, or in situ cervical cancer - History of any prior gastrointestinal conditions (e.g., diarrhea syndromes, inflammatory bowel disease) that may have affected the absorption or metabolism of study intervention; participants with prior gastric bariatric interventions (e.g., Lap‑Band) which were reversed were not excluded - History of acute hepatitis within 6 months of screening or chronic hepatitis (including nonalcoholic steatohepatitis), anti-HIV Type 1 and 2 antibody, HBsAg, or anti‑hepatitis C antibody testing at screening - History of spinal surgery or a history of brain or spinal cord abnormality (CSF collection subset only) - Presence of infection at the puncture site or spinal deformities as noted on spinal X‑rays that would have interfered with the lumbar puncture procedure (CSF collection subset only) - History of recent (within the last month) lumbar puncture and collection of CSF prior to the screening date of the study (CSF collection subset only) - Recent spinal X-rays (screening X-ray if no X-rays within 12 months prior to screening were available) with evidence to suggest that abnormalities would have interfered with the lumbar puncture procedure (CSF collection subset only) |

**Table S1** (continued)**.** Study enrollment criteria.

| **Exclusion criteria** (continued) | |
| --- | --- |
| Medical conditions and history (continued) | - Known hypersensitivity to lidocaine or its derivatives used during CSF collection or any medication used to prepare the skin prior to the lumbar puncture (CSF collection subset only) - History or current condition of chronic lower back pain (CSF collection subset only) - Had known or suspected coagulopathy, prothrombin time/activated partial thromboplastin time/international normalized ratio values significantly outside the normal laboratory range, or significantly decreased platelet count at the discretion of the investigator (CSF collection subset only) - Used anticoagulant (e.g., NSAIDs, heparin, or heparin derivatives) within 10 days prior to lumbar puncture (CSF collection subset only) - Clinically significant abnormalities (as determined by the investigator) in physical examination or laboratory safety test at screening as per guidelines below: - ALT or AST greater than the ULN OR - Total bilirubin greater than 1.5 mg/dL (except for patients with a diagnosis of Gilbert’s disease) OR - Serum albumin less than 2.8 g/dL - History of alcohol or other substance abuse within the previous 5 years - Any clinical condition or previous surgery that might affect the absorption, distribution, biotransformation, or excretion of ubrogepant, erenumab or galcanezumab - Known allergy or sensitivity to the study intervention or its components or other anti-CGRP medications |
| Prior/concomitant therapy | - Requirement for any prohibited medication that could not be discontinued or switched to an allowable, alternative medication. Participants who required daily intake of antacids, proton pump inhibitors, and histamine H2 antagonists were excluded - Certain medications were allowed but required stable dosing before screening |
| Prior/concomitant clinical study experience | - Previously participated in an investigational study of ubrogepant - Participation in any other clinical investigation using an experimental drug within 30 days prior to study intervention administration - Participation in a blood or plasma donation program within 60 or 30 days, respectively, prior to study intervention administration |
| Diagnostic assessments | - Sitting systolic BP ≥160 mmHg or ≤90 mmHg or sitting diastolic BP ≥100 mmHg or ≤50 mmHg at the Screening Visit. - Abnormal ECG results thought to be PCS according to the investigator or designee, or QT prolongation (QTcF ≥450 msec for male participants or ≥470 msec for female participants or uncorrected QT ≥500 msec) at the Screening Visit. - Abnormal and clinically significant results according to the investigator or designee, on physical examination, medical history, hematology, clinical chemistry, or urinalysis |

**Table S1** (continued)**.** Study enrollment criteria.

| **Exclusion criteria** (continued) | |
| --- | --- |
| Other | - Participants must not have consumed Seville oranges, beverages or food containing quinine (bitter lemon, tonic water), poppy seeds, or taken dietary supplements or other foods or beverages that may have affected various drug-metabolizing enzymes and transporters (e.g., grapefruit, grapefruit juice, grapefruit-containing beverages), vegetables from the mustard green family (e.g., kale, broccoli, watercress, collard greens, kohlrabi, brussel sprouts, mustard), or charbroiled meats within 14 days prior to dosing and throughout the duration of the study. - Employee, or immediate relative of an employee, of the sponsor, any of its affiliates or partners, or the study center - Breastfeeding |

**Table S2.** Listing of TEAEs in participants who received a single dose of 100 mg ubrogepant and underwent a single post-dose collection of CSF.

| **Participant** | **Preferred Term** | **Study day** | **TEAE**  **Severity** | **Related to study intervention** | **Outcome** |
| --- | --- | --- | --- | --- | --- |
| 1 | Puncture site pain^a^ | 1 | Mild | No | Resolved |
| 2 | No TEAEs reported | --- | --- | --- | --- |
| 3 | Puncture site pain | 1 | Mild | No | Resolved |
|  | Post-lumbar puncture syndrome | 1 | Mild | No | Resolved |
|  | Neuropathy peripheral^b^ | 1 | Mild | No | Resolved |
| 4 | Puncture site pain  Neck pain | 1  2 | Mild  Mild | No  No | Resolved  Resolved |
| 5 | Post-lumbar puncture syndrome | 2 | Mild | No | Resolved |
| 6 | Puncture site pain | 2 | Mild | No | Resolved |
|  | Vomiting | 2 | Mild | No | Resolved |
| 7 | No TEAEs reported | --- | --- | --- | --- |
| 8 | Puncture site pain | 1 | Mild | No | Resolved |

^a^Puncture site pain refers to pain at the site of lumbar puncture. ^b^IV site neuropathy (left wrist). No serious TEAEs or deaths occurred. TEAE, treatment-emergent adverse event (≤30 days after ubrogepant dosing).
